# Regulation of Δ6Fads2 Gene Involved in LC-PUFA Biosynthesis Subjected to Fatty Acid in Large Yellow Croaker (*Larimichthys crocea*) and Rainbow Trout (*Oncorhynchus mykiss*)

**DOI:** 10.3390/biom12050659

**Published:** 2022-04-30

**Authors:** Jie Sun, Jingqi Li, Yongnan Li, Jianlong Du, Nannan Zhao, Kangsen Mai, Qinghui Ai

**Affiliations:** 1 The Key Laboratory of Aquaculture Nutrition and Feed (Ministry of Agriculture and Rural Affairs), The Key Laboratory of Mariculture (Ministry of Education), Ocean University of China, 5 Yushan Road, Qingdao 266003, China; sj2878@stu.ouc.edu.cn (J.S.); lijq04@haid.com.cn (J.L.); lyn@qdio.ac.cn (Y.L.); dujianlong@ouc.edu.cn (J.D.); 22320200155986@stu.xmu.edu.cn (N.Z.); kmai@ouc.edu.cn (K.M.); 2 Laboratory for Marine Fisheries Science and Food Production Processes, Qingdao National Laboratory for Marine Science and Technology, 1 Wenhai Road, Qingdao 266237, China

**Keywords:** Δ6Fads2, LC-PUFA, transcriptional regulation, C/EBPα, GATA3

## Abstract

Δ6 fatty acyl desaturase (Δ6Fads2) is regarded as the first rate-limiting desaturase that catalyzes the biosynthesis of long-chain polyunsaturated fatty acids (LC-PUFA) from 18-carbon fatty acid in vertebrates, but the underlying regulatory mechanism of *fads2* has not been comprehensively understood. This study aimed to investigate the regulation role of *fads2* subjected to fatty acid in large yellow croaker and rainbow trout. In vivo, large yellow croaker and rainbow trout were fed a fish oil (FO) diet, a soybean oil (SO) diet or a linseed oil (LO) diet for 10 weeks. The results show that LO and SO can significantly increase *fads2* expression (*p* < 0.05). In vitro experiments were conducted in HEK293T cells or primary hepatocytes to determine the transcriptional regulation of *fads2.* The results show that CCAAT/enhancer-binding protein α (C/EBPα) can up-regulate *fads2* expression. GATA binding protein 3 (GATA3) can up-regulate *fads2* expression in rainbow trout but showed opposite effect in large yellow croaker. Furthermore, C/EBPα protein levels were significantly increased by LO and SO (*p* < 0.05), *gata3* expression was increased in rainbow trout by LO but decreased in large yellow croaker by LO and SO. In conclusion, we revealed that FO replaced by LO and SO increased *fads2* expression through a C/EBPα and GATA3 dependent mechanism in large yellow croaker and rainbow trout. This study might provide critical insights into the regulatory mechanisms of *fads2* expression and LC-PUFA biosynthesis.

## 1. Introduction

Long-chain polyunsaturated fatty acids (LC-PUFA) such as eicosapentaenoic acid (EPA; 20:5n-3) and docosahexaenoic acid (DHA; 22:6n-3) have been identified to play important roles in multiple physiological functions including nervous development [1,2], cardiovascular diseases [3,4], and anti-inflammatory effects [5,6]. There are two ways vertebrates can acquire LC-PUFA; one is to get LC-PUFA from diets and the other is to synthesize it endogenously from shorter PUFA, namely linoleic acid (LA; 18:2n-6) and α-linolenic acid (ALA; 18:3n-3), through a series of desaturation and elongation reactions [6,7]. However, the ability to synthesize LC-PUFA endogenously varies among species. Stable isotope tracer studies in human indicated that the conversion of ALA to LC-PUFA, particularly DHA, is limited [8]. The capability to synthesize LC-PUFA from LA and ALA is also limited in marine fish; however, freshwater fish and salmonid species have the capability to synthesize LC-PUFA [9,10,11]. To date, the differential LC-PUFA biosynthesis across species is regarded as a result of different Δ6 fatty acyl desaturase (Δ6Fads2) activities by many researchers [12]. Δ6Fads2 is the first and a rate-limiting enzyme in the process of LC-PUFA biosynthesis from precursor fatty acids [12]. Δ6Fads2 is encoded by the *fads2* gene in mammals and teleosts [13,14], and it is responsible for the first desaturase step of converting LA and ALA to longer carbon chain fatty acids such as arachidonic acid (ARA), EPA, and DHA. However, the regulatory mechanism underlying *fads2* remains poorly understood.

In mammals, it is known that the activities of fatty acids desaturases could be affected by diets and hormones [15]. A study in rats has given evidence that Δ6Fads2 activities could be lowered by dietary LC-PUFA [15]. Similar results were also identified in fishes such as large yellow croaker (*Larimichthys crocea*) [16], Japanese seabass (*Lateolabrax japonicus*) [17], and grouper larvae (*Epinephelus coioides*) [18]. The expression of *fads2* could be up-regulated by vegetable oils which are rich in 18-carbon fatty acids, such as LA and ALA [17,19]. So far, many studies indicated that the mRNA levels of *fads2* were varied among fish species when in response to dietary fatty acids [16,17,18,20]. The dramatic change in gene expression that accompanies physiological and biochemical reactions of specialized cellular function is controlled mainly at the transcriptional level. Therefore, it is important to obtain the factors which play crucial roles in *fads2* transcription. 

C/EBPα (CCAAT/enhancer-binding protein α) is the first member of the C/EBP family of the basic region leucine zipper proteins which is capable of binding eukaryotic gene promoters and regulating gene transcription [21,22]. Many studies have indicated that the C/EBPα is involved in many processes such as cell differentiation [23,24], lipid deposition [25,26], fatty acid metabolism [27], and energy metabolism [28]. Dong et al. (2016) suggested that C/EBP proteins could modulate the activities of Δ4Fads2 promoter and be involved in the process of DHA biosynthesis mediated by the Δ4Fads2 in rabbitfish (*Siganus canaliculatus*). GATA binding protein 3 (GATA3) belongs to the GATA family of transcription factors, which have been implicated as being involved in the regulation of cell fate and differentiation in various cell types [29,30]. To date, the function of C/EBPα and GATA3 in *fads2* transcription and LC-PUFA biosynthesis has not been studied.

In the present study, a marine teleost (large yellow croaker) and a freshwater teleost, (rainbow trout (*Oncorhynchus mykiss*)), which are both important farming fish species in China, were used as experimental model animals. It has been verified that large yellow croaker has a limited capability to synthesize LC-PUFA as compared to rainbow trout. Hence, they represent suitable model species for the study of LC-PUFA biosynthesis. *Fads2* plays an important role in LC-PUFA biosynthesis, but the underlying regulatory mechanism of *fads2* in large yellow croaker and rainbow trout has not been comprehensively understood. Therefore, this study was designed to explore the transcriptional regulation of *fads2* subjected to fatty acid and its differences between large yellow croaker and rainbow trout in order to provide critical insights into the role of molecular regulatory mechanisms in *fads2* expression and LC-PUFA biosynthesis.

## 2. Materials and Methods

### 2.1. Animal Studies

A 10-week feeding experiment was carried out using 3 purified diets. The diets were formulated with fish oil, soybean oil, and linseed oil as the main lipid sources, the formulation and proximate composition are listed in Supplementary Table S1, and fatty acid composition is listed in Section 3.1. A total of 1080 large yellow croakers (15.42 ± 0.01 g mean weight) were randomly distributed into 9 floating cages (1.5 m × 1.5 m × 2 m), and each diet was randomly assigned to cages in triplicate. During the experiment period, the fish were fed to apparent satiation twice daily (at 6:00 and 18:00), the temperature ranged from 24 to 28 °C, the salinity ranged from 26 to 29‰, pH ranged from 7.4 to 7.8 and dissolved oxygen ranged from 6 to 7 mg/L. Rainbow trout were farmed in 60 L tanks as the previous study reported [31] with a continuous flow of fresh water. In brief, a total of 315 rainbow trout (11.03 ± 0.02 g mean weight) were randomly distributed into 9 tanks, and each diet was randomly assigned to tanks in triplicate. During the experimental period, the water temperature was kept at 18 ± 1 °C. At the end of feeding trials, livers, muscle, and intestines of large yellow croaker and rainbow trout were collected from 3 individuals in each cage and stored at −80 °C for RNA extraction, Western blotting, and fatty acid composition analysis.

### 2.2. Fatty Acid Composition Analysis 

The fatty acid composition was determined according to the protocol as a previous study reported [32]. Briefly, the total lipid of fish tissues (liver, muscle, and intestine) was extracted using chloroform and methanol in a 2:1 ratio, and fatty acids in the samples were esterified into fatty acid methyl esters (FAME) by KOH-ethanol and methanolic hydrogen chloride in 75 to 80 °C for 20 min respectively. A previous test has been conducted to make sure that all fatty acids can be esterified following the procedures above. At last, 1 mL hexane was added to the mixture above, shaken vigorously for 1 min, and then allowed to separate into two layers. FAME in the upper layer was separated and quantified by an HP6890 gas chromatograph (Agilent Technologies Inc., Santa Clara, CA, USA) with a fused silica capillary column (007-CW, Hewlett Packard, Palo Alto, CA, USA) and a flame ionization detector. Nitrogen was used as carrier gas and the column temperature was programmed to rise from 150 to 200 °C at a rate of 15 °C/min, from 200 to 250 °C at a rate of 2 °C/min. The injector and detector temperature was 250 °C, respectively. Each methyl ester was identified by comparison to the standard. Results are expressed as the percentage of each fatty acid to the total fatty acid.

### 2.3. RNA Extraction and cDNA Synthesis

Prior to RNA extraction, tissues were ground to powder in liquid nitrogen. The samples were added to the Trizol reagent (Takara, Dalian, China). Subsequently, total RNA was extracted following the manufacturer’s protocol. The integrity of RNA was detected by electrophoresis using 1.2% denatured agarose gel. The cDNA was reverse transcribed from RNA by PrimeScript RT reagent kit (Takara, Dalian, China) following the protocol supplied by the manufacturer.

### 2.4. Absolute Quantification PCR (AQ-PCR), RT-qPCR and Western Blotting

The mRNA expression levels were quantified in a quantitative thermal cycler Mastercycler ep realplex (Eppendorf, Hamburg, Germany) with SYBR Green real-time PCR kit (Takara, China). Absolute quantification of PCR and RT-qPCR was carried out according to the method as previously described [31,33]; the primers used are listed in Supplementary Table S2. As for RT-qPCR, *β-actin*, *gapdh*, and *rpl17* were selected to test their suitability for the normalization of gene expression levels in various tissues of large yellow croaker and rainbow trout. No significant differences in *β-actin* expression were detected among all treatments, suggesting that *β-actin* could be used as a reference gene. Further, the mRNA expression of each gene was normalized to that of *β-actin* using the 2^−ΔΔct^ method [34]. Western blotting was carried out according to previously described the methods [35,36]. The primary antibodies used in this study were against FLAG (Cell Signaling Technology, Danvers, MA, USA) and C/EBPα (Abcam, Cambridge, UK).

### 2.5. Primary Culture of Hepatocytes and Incubation 

In order to simulate the in vivo experiments and further verify the results, we carried out in vitro experiments. The methods of primary culture of hepatocytes of rainbow trout were described previously [37]. Hepatocytes were seeded into 6-well plates at a density of 2 × 10^6^ cells/ml and were maintained in a DMEM/F12 medium (BI, Kibbutz Beit-Haemek, Beit HaEmek, Israel) containing 15% FBS (BI, Kibbutz Beit-Haemek, Israel) and incubated at 18 °C. Microscopic examination ensured that hepatocytes progressively reassociated throughout the culture to form two-dimensional aggregates, in agreement with earlier reports [38,39]. As for the large yellow croaker, hepatocytes were isolated from 5 individuals (50 g), which were starved for 24 h according to the methods previously described [40]. Hepatocytes were seeded into 6-well plates at a density of 2 × 10^6^ cells/mL and were maintained in DMEM/F12 medium (BI, Kibbutz Beit-Haemek, Israel) containing 15% FBS (BI, Kibbutz Beit-Haemek, Israel) and antibiotics in a 5% CO_2_ atmosphere at 28 °C. The hepatocytes were incubated with ALA, LA, EPA, and DHA to confirm the effect of fatty acids on the transcription of the *fads2* in vitro. The solvent group was set as the control group. The fatty acid (ALA, LA, EPA, and DHA, Cayman Chemical Co., Ann Arbor, MI, USA) was supplemented to cells on 6-well plates in the form of BSA/fatty acid complexes that were prepared at 100 μM concentration, incubated for 12 h. After incubation, cells were lysed in the wells and harvested for RNA extraction.

### 2.6. Genomic DNA Extraction, fads2 Promoter Cloning, and Deletion Mutant Construction

Large yellow croaker and rainbow trout genomic DNA was extracted by DNAiso Reagent (Takara, Dalian, China) according to the manufacturer’s instructions. Primers used to clone the promoters were designed according to the promoter sequence of *fads2* which had been cloned by our laboratory. PCR was conducted using *TransStart FastPfu Fly DNA Polymerase* (TransGen Biotech, Beijing, China) to get the promoter of *fads2* of large yellow croaker (996 bp). Then, the promoter was subcloned into pEASY-T1 vector (TransGen Biotech, Beijing, China) for sequencing. The recombinant pEASY-T1 plasmid with the right sequence was used as the template for following PCR cloning. To identify the core promoter region of *fads2* of large yellow croaker, a deletion mutant assay was conducted. The recombinant pEASY-T1 plasmid was used as a template for PCR cloning with different primers (Supplementary Table S3) to get different length products with *XbaI* and *KpnI* cleavage sites, and 7 DNA fragments were obtained. The products of PCR were recombined to pEASY-T1 plasmid and digested by restriction endonuclease *XbaI* and *KpnI* (Takara, Dalian, China). Then, the 7 obtained DNA fragments were inserted into the pGL3.0-basic firefly luciferase reporter vector which has no promoter. The constructed reporter vectors were named as LD6D1, LD6D2, LD6D3, LD6D4, LD6D5, LD6D6, and LD6D7. The reporter vectors named OD6D1, OD6D2, OD6D3, OD6D4, OD6D5, OD6D6, and OD6D7 in rainbow trout were obtained through the same methods in large yellow croaker. All plasmids were confirmed by DNA sequencing and prepared using an EasyPure HiPure Plasmid MiniPrep kit (TransGen Biotech, Beijing, China). 

### 2.7. Cell Culture, Transfection, and Luciferase Reporter Assay

The human embryonic kidney cell line (HEK293T cell line) was cultured in high-glucose DMEM supplemented with 10% FBS (Biological Industries), 100 U/mL penicillin, and 100 μg/mL streptomycin at 37 °C and 5% CO_2_. Cells were inoculated into 24-well cell culture plates at 2 × 10^5^ cells per well and cultured for 24 h to 80–90% confluence before being transfected. To determine the promoter activities, HEK293T cells were co-transfected with deletion mutational recombinant vectors or pGL3-Basic and phRL-CMV plasmid using lipofectamine 2000 (Invitrogen, Carlsbad, CA, USA) according to the manufacturer’s instructions. To confirm the effects of transcription factors (all used expression plasmid were stored in our laboratory) on the *fads2* promoter, HEK293T cells were co-transfected with the *fads2* promoter, expression plasmids, and phRL-CMV plasmid. The promoter-less pGL3-basic vector was used as a negative control for each transfection assay. All assays were performed with 3 independent transfections. The luciferase activity was measured using a TransDetect double-luciferase reporter assay kit (TransGen Biotech, Beijing, China).

### 2.8. RNA Interference Assay

Small interference RNAs (siRNA) were designed and synthesized for gene knockdown according to the C/EBPα and GATA3 sequence by GenePharma Biotech (Shanghai, China). C/EBPα and GATA3 gene knockdown was performed using Xfect RNA Transfection Reagent (TaKaRa, Dalian, China) according to the protocol supplied by the manufacturer. 

### 2.9. Site-Directed Mutation

To determine the potential functions of C/EBPα and GATA3 sites on the promoter activity of large yellow croaker and rainbow trout, site-directed mutation of recombinant plasmids was carried out by PCR reaction with the mutation sites designed in the primers (Supplementary Table S4). The recombinant plasmids, LD6D1 and OD6D1, were regarded as wild-type and a template for the construction of mutational recombinant plasmids, respectively. Site-directed mutational recombinant plasmids and over-expression plasmids were co-transfected into the HEK293T cell line for dual-luciferase reporter assay. 

### 2.10. Chromatin Immunoprecipitation (ChIP) Assay

The *fads2* promoter and expression plasmids (pCS2+ -C/EBPα-flag or pCS2+ -GATA3-flag) were co-transfected into HEK293T cells at a ratio of 1:2. After 24 h of transfection, the HEK293T cells were cross-linked with 1% formaldehyde at 37 °C for 10 min. Protein lysates collected from these cells were incubated with anti-IgG or anti-flag antibodies, then they were analyzed using ChIP assay as per ChIP Kit protocol (Beyotime Institute of Biotechnology, Shanghai, China). Bound target DNA fractions were analyzed by PCR with the specific primers (Supplementary Table S2) containing the binding sites of C/EBPα and GATA3. PCR products were electrophoresed in 1.2% agarose gels and detected by Gel stain (TransGen Biotech, Beijing, China). 

### 2.11. Electrophoretic Mobility Shift Assay (EMSA)

The nuclear protein was prepared using NE-PER Nuclear and Cytoplasmic Extraction Reagents (Thermo Fisher Scientific, Waltham, MA, USA). The C/EBPα sequence in the *fads2* promoter region of large yellow croaker and rainbow trout with 5′ biotin-labeled probes was synthesized by Sangon Biotech (Shanghai, China). The sequences were as follows: forward, 5′-CATGCTTCATGTCGAAACAGACAAATGATG-3′, and reverse, 5′-CATCATTTGTCTGTTTCGACATGAAGCATG-3′ of large yellow croaker; forward, 5′-AGAGTCACACCATTTCACCACGTAGGTGTG-3′, and reverse, 5′-CACACCTACGTGGTGAAATGGTGTGACTCT-3′ of rainbow trout. The sequences of the competitive probes were identical to those of the biotin-labeled probes, but there was no biotin-labeled at each end. The sequences of mutative competitive probes were as follows: forward, 5′-CATGCTTCATGTCACGACAGACAAATGATG-3′, and reverse, 5′-CATCATTTGTCTGTCGTGACATGAAGCATG-3′ of large yellow croaker; forward, 5′-AGAGTCACACGAGCTATCTACGTAGGTGTG-3′, and reverse, 5′-CACACCTACGTAGATAGCTCGTGTGACTCT-3′ of rainbow trout. The Annealing Buffer for DNA Oligos (Beyotime Institute of Technology, Shanghai, China) was adopted for DNA oligonucleotide annealing according to the manufacturer’s instructions. The annealed probes were stored at −20 °C until use. According to the instructions of the LightShift Chemiluminescent EMSA Kit (Thermo Fisher Scientific, CA, USA), binding reactions and chemiluminescent detection were conducted. For the competition experiment, a 40-fold molar excess of the unlabeled probe was included in the preincubation mixture before the addition of the labeled probe. 

### 2.12. Statistical Analysis 

All data were presented as means ± SEMs. The data on fatty acid composition and Western blotting were analyzed by one-way ANOVA with the help of SPSS 19.0 followed by Tukey’s multiple comparison test. The data of dual-luciferase trial and site-directed mutation were analyzed by independent *t*-test. The data of absolute quantification PCR were analyzed by both one-way ANOVA and independent *t*-test. *p* < 0.05 was applied as a significant difference and *p* < 0.01 as a highly significant difference.

## 3. Results

### 3.1. Fatty Acid Compositions in Liver, Muscle, and Intestine of Large Yellow Croaker and Rainbow Trout

The diet was rich in ALA, LA, DHA, and EPA in linseed oil (LO), soybean oil (SO), and fish oil (FO) diet, respectively. The fatty acid composition of tissues reflected the fatty acid composition of the diet (Table 1 and Table 2). FO substituted by LO and SO significantly increased the relative content of 18-carbon fatty acids (*p* < 0.05), such as C18:2n-6 and C18:3n-3 in the liver, muscle, and intestine of large yellow croaker and rainbow trout. The relative content of LC-PUFA in tissues of large yellow croaker and rainbow trout fed LO and SO was significantly lower than the fish fed FO (*p* < 0.05) except in the liver of rainbow trout. The data of the diet and liver/muscle in large yellow croaker were already published in our previous study [41].

### 3.2. Absolute Quantitative Analysis of fads2 Expression 

**Figure 1:**
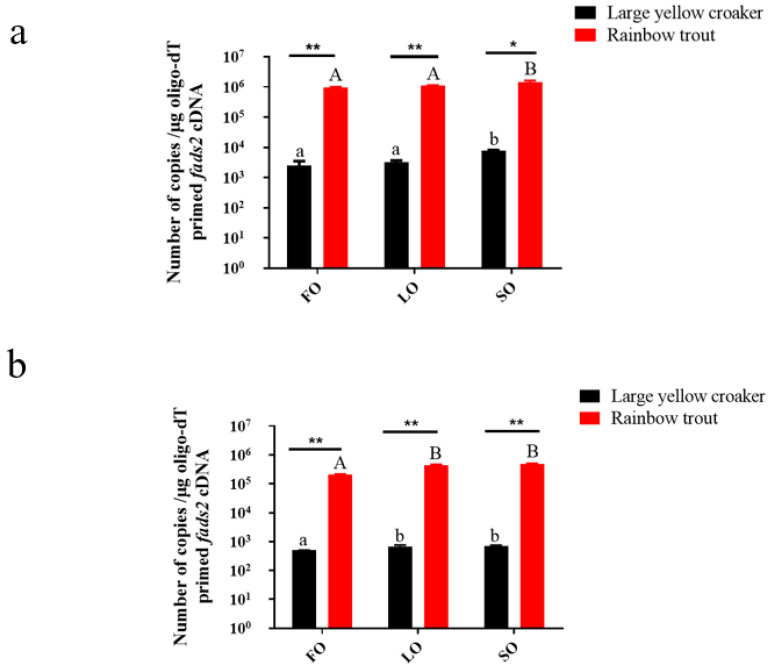
The absolute expression of *fads2* in liver (**a**) and intestine (**b**) of large yellow croaker and rainbow trout. Values are presented as mean ± SEM (*n* = 3). The “*” means a significant difference (*p* < 0.05) and “**” means a highly significant difference (*p* < 0.01). Bars without sharing a common letter are significantly different (*p* < 0.05). FO: 100% fish oil as a lipid source in the diet. LO: 100% linseed oil as a lipid source in the diet. SO: 100% soybean oil as a lipid source in the diet.

Tissues collected from the feeding experiment showed that the fads2 expression was up-regulated significantly by LO and SO (Figure 1). In large yellow croaker, gene expression in the intestine was significantly up-regulated by SO and LO (*p* < 0.05), and gene expression in the liver was also significantly up-regulated by SO (*p* < 0.05). The results were the same in rainbow trout. Furthermore, the fads2 expression in rainbow trout was higher than in large yellow croaker. To be more specific, in the liver, the gene expression in rainbow trout had a highly significant difference in FO and LO group (*p* < 0.01) and a significant difference in the SO group (*p* < 0.05) compared with large yellow croaker (Figure 1a). In the intestine, the gene expression in rainbow trout had a highly significant difference in the FO, LO, and SO groups (*p* < 0.01) compared with large yellow croaker (Figure 1b).

### 3.3. Relative fads2 Expression in Hepatocytes of Large Yellow Croaker and Rainbow Trout in Response to Fatty Acids

**Figure 2:**
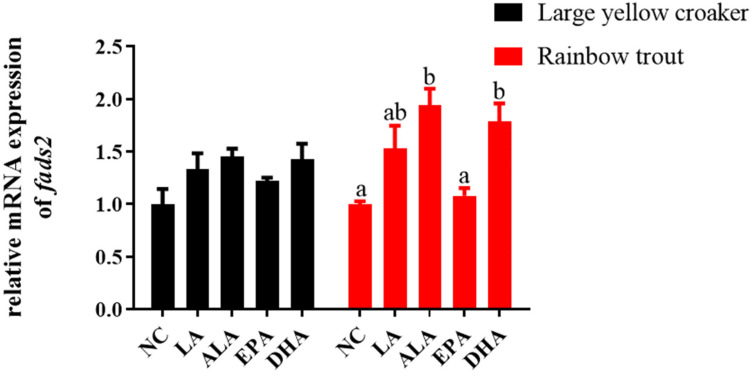
The relative expression of *fads2* in hepatocytes of large yellow croaker and rainbow trout. The hepatocytes were in response to different fatty acids incubation of 100 μM concentration for 12 h. Values are presented as mean ± SEM (*n* = 3). NC: negative control, LA: linoleic acid, ALA: α-linolenic acid, EPA: eicosapentaenoic acid, DHA: docosahexaenoic acid. Bars without sharing a common letter are significantly different (*p* < 0.05).

In order to further verify the in vivo experiments, the hepatocytes were incubated with ALA, LA, EPA, and DHA to confirm the influence of fatty acids on the transcription of the *fads2* in vitro. Compared with the control group, the expression level of *fads2* had no significant differences in large yellow croaker (*p* > 0.05) (Figure 2). The *fads2* expression was significantly increased in the ALA and DHA groups (*p* < 0.05), and had no significant differences in the LA and EPA groups (*p* > 0.05) in rainbow trout (Figure 2).

### 3.4. Analysis of Deletion Mutants’ Activities

**Figure 3:**
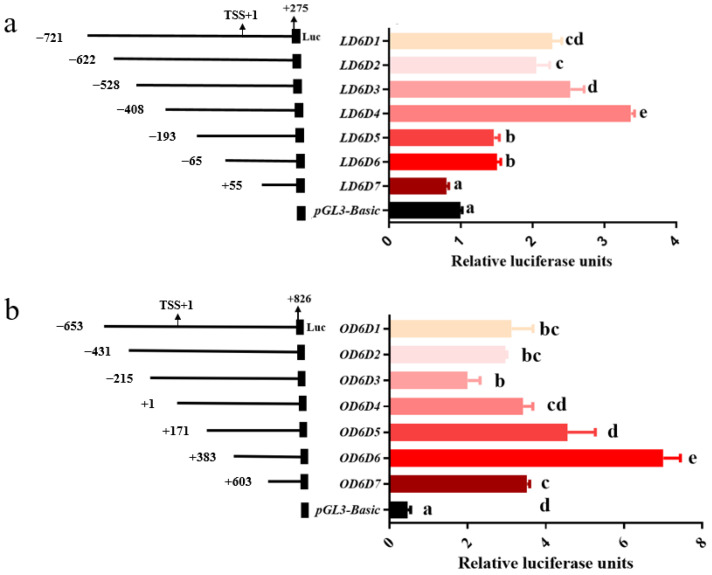
The deletion analysis of *fads2* promoter of large yellow croaker (**a**) and rainbow trout (**b**). Deletion mutant constructs are represented on the left. Sequences are numbered relative to the putative transcription start site. Luciferase coding by closed boxes. Promoter activities are represented on the right. The values represent normalized activity (Firefly luciferase/Renilla luciferase) relative to the pGL3-Basic (as a control). TSS: transcription start site. Bars without sharing a common letter are significantly different (*p* < 0.05).

Gene expression is controlled mainly at the transcription level, where the promoter plays an important role. To determine the core promoter fragments that were able to regulate the basal transcription of *fads2*, seven recombinant reporter vectors of large yellow croaker and rainbow trout contained different promoter sequence length were constructed respectively. The activities of progressive deletion mutants were analyzed by dual-luciferase reporter assays. As the results suggested (Figure 3), the activities of the promoters had dramatic changes with the progressive deletion. Fragments (−622 bp to −529 bp, relative to the transcription start site (TSS)) and (−528 bp to −409 bp) in large yellow croaker and fragment (−215 bp to +383 bp) in rainbow trout caused a gradual increase in the activities of the promoters. Fragments (−408 bp to −194 bp) and (−65 bp to +54 bp) in large yellow croaker and fragment (+383 bp to +826 bp) in rainbow trout caused a significant decrease in the activities of the promoters. Indicated that the fragments (−408 bp to −194 bp) and (−65 bp to +54 bp) in large yellow croaker and fragment (+383 bp to +826 bp) in rainbow trout were the core promoter regions.

### 3.5. Transcriptional Regulation of the fads2 Promoter by Transcription Factors

**Figure 4:**
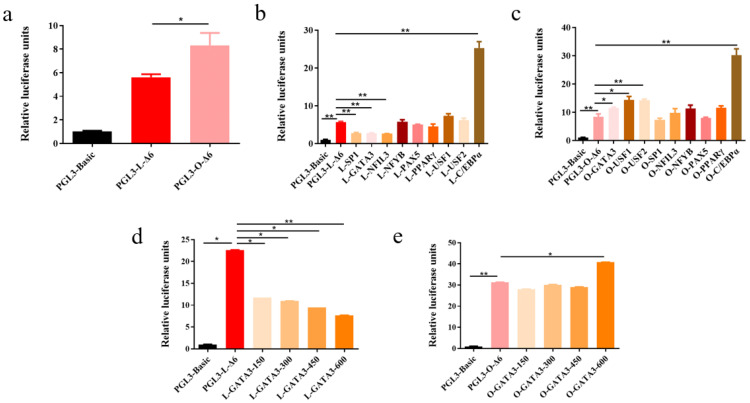
Transcriptional regulation of *fads2* promoter in large yellow croaker and rainbow trout. The comparison of *fads2* promoter activity between large yellow croaker and rainbow trout (**a**). The influence of transcription factors over-expression on *fads2* promoter activity in large yellow croaker (**b**) and rainbow trout (**c**). Transcriptional regulation of *fads2* promoter by different concentrations of GATA3 in large yellow croaker (**d**) and rainbow trout (**e**). Values are presented as mean ± SEM (*n* = 3). The “*” means a significant difference (*p* < 0.05) and “**” means a highly significant difference (*p* < 0.01).

Several transcription factors including CEBPα, GATA3, SP1, PPARγ, NFIL3, NFYB, PAX5, USF1, and USF2 were predicted to bind to the *fads2* promoter region of large yellow croaker and rainbow trout by the bioinformatics software, including JASPAR, TRANSFAC, and TF binding. The predicted sites of these transcription factors were widely distributed in the *fads2* promoter region. To determine the roles of these transcription factors in regulating *fads2* promoter activity in large yellow croaker and rainbow trout, the expression vectors and reporter vectors, LD6D1 (PGL3-L-Δ6)/OD6D1 (PGL3-O-Δ6), were co-transfected into HEK293T cells and the dual-luciferase assay was conducted, using pGL3-Basic and pCS2+ as controls. The results showed that the activity of rainbow trout *fads2* promoter was significantly higher than large yellow croaker (*p* < 0.05) (Figure 4a). The transcription factor SP1 and NFIL3 could highly significantly down-regulate *fads2* promoter activity in large yellow croaker (*p* < 0.01) but had no regulatory activity on *fads2* promoter of rainbow trout. The transcription factor USF1 and USF2 could significantly up-regulate *fads2* promoter activity in rainbow trout (*p* < 0.05) but had no regulatory activity on *fads2* promoter of large yellow croaker. The transcription factor NFYB, PAX5, and PPARγ had no regulatory activity on *fads2* promoter of both large yellow croaker and rainbow trout. The transcription factor C/EBPα could highly significantly up-regulate *fads2* promoter activity in both large yellow croaker and rainbow trout (*p* < 0.01). The transcription factor GATA3 could highly significantly down-regulate *fads2* promoter activity in large yellow croaker (*p* < 0.01) but up-regulate the activity in rainbow trout (*p* < 0.05) (Figure 4b,c). The effect of GATA3 was increased with the increase of GATA3 concentration (Figure 4d,e).

### 3.6. Absolute Quantitative Analysis of c/ebpα and gata3 Expression, and Relative Expression of C/EBPα Protein Level

**Figure 5:**
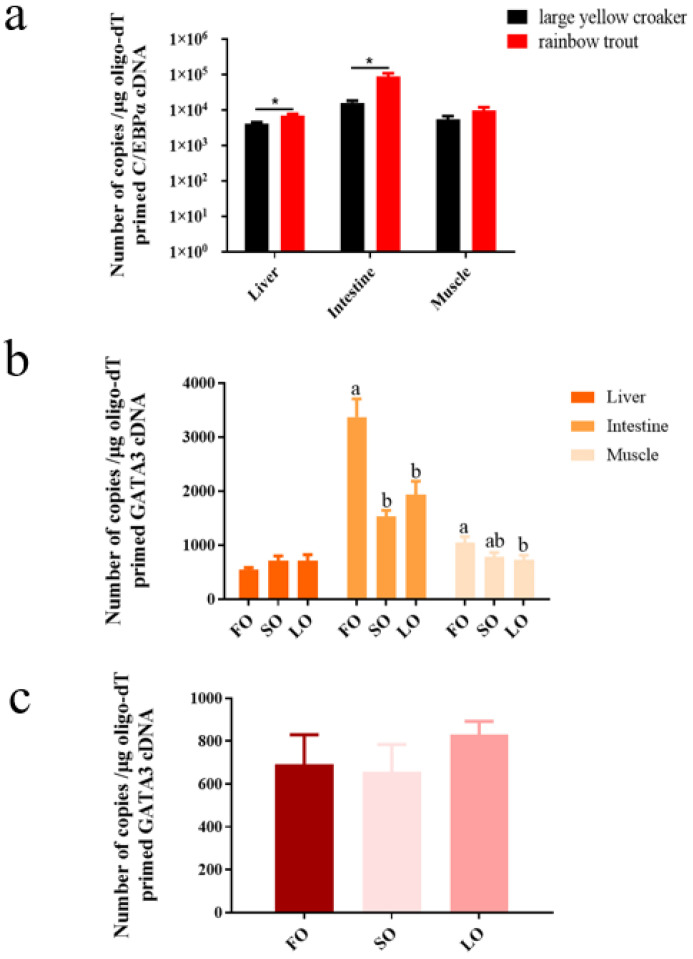
The absolute expression of C/EBPα and GATA3 in tissues of large yellow croaker and rainbow trout. The absolute expression of C/EBPα gene in liver, intestine, and muscle of large yellow croaker and rainbow trout (**a**). The absolute expression of GATA3 gene in liver, intestine, and muscle of large yellow croaker (**b**) and liver of rainbow trout (**c**). Values are presented as mean ± SEM (*n* = 3). The “*” means a significant difference (*p* < 0.05). Bars without sharing a common letter are significantly different (*p* < 0.05). FO: 100% fish oil as a lipid source in the diet. LO: 100% linseed oil as a lipid source in the diet. SO: 100% soybean oil as a lipid source in the diet.

**Figure 6:**
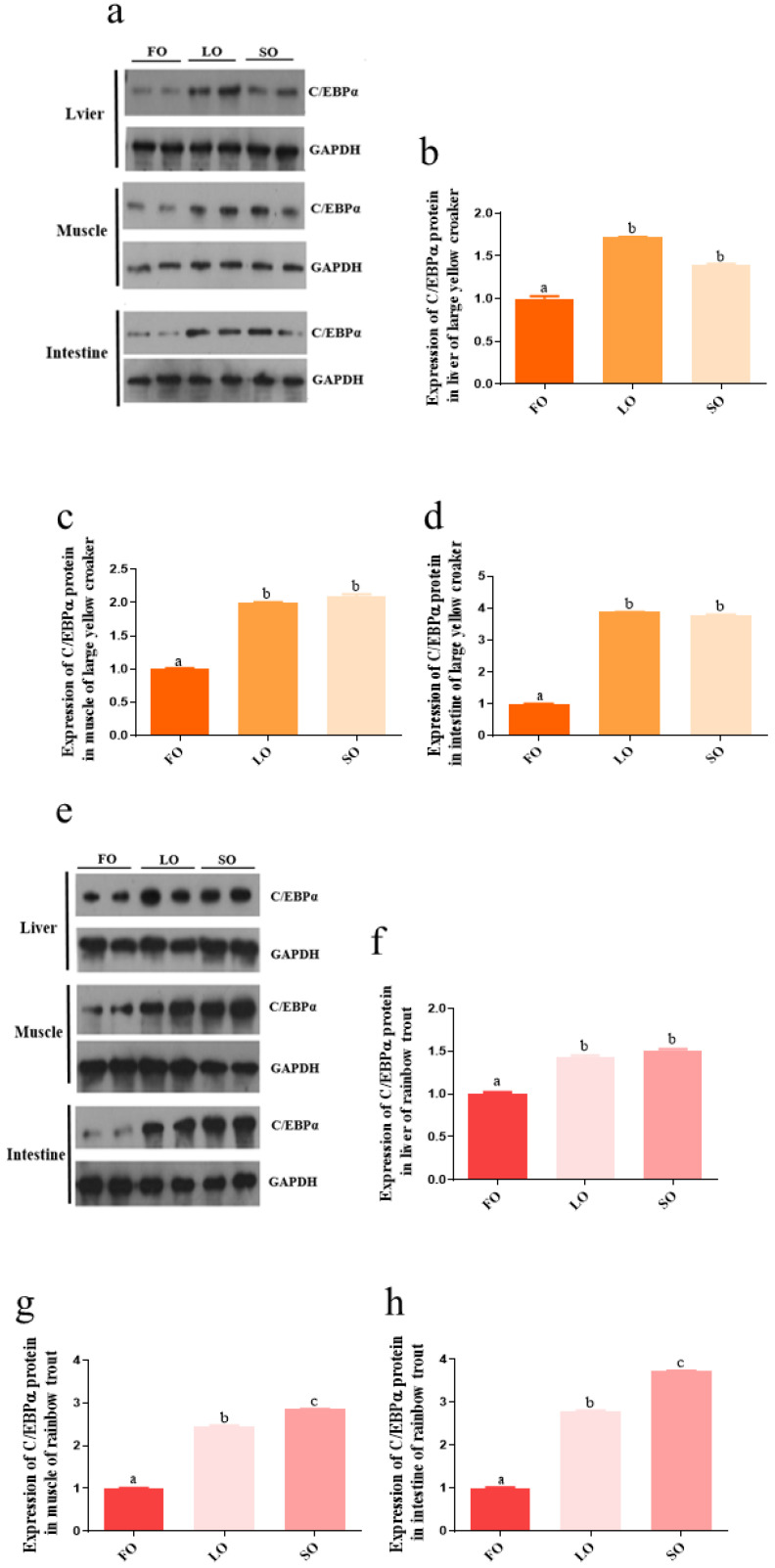
The relative expression of C/EBPα protein in tissues of large yellow croaker (**a**–**d**) and rainbow trout (**e**–**h**). Bars without sharing a common letter are significantly different (*p* < 0.05). FO: 100% fish oil as a lipid source in the diet. LO: 100% linseed oil as a lipid source in the diet. SO: 100% soybean oil as a lipid source in the diet.

The transcription factor C/EBPα could highly significantly up-regulate *fads2* promoter activity in both large yellow croaker and rainbow trout. Transcription factor GATA3 was the only one that could significantly up-regulate the promoter activity in rainbow trout but down-regulate the promoter activity in large yellow croaker. It seemed to be one of the reasons for the difference in *fads2* expression between large yellow croaker and rainbow trout. In order to further investigate the function of C/EBPα and GATA3, we first conducted the absolute quantitative analysis and Western blotting. It was obvious that the *c/ebpα* expression in the liver (*p* < 0.05) and intestine (*p* < 0.05) of rainbow trout was higher than in large yellow croaker (Figure 5a). The protein level of C/EBPα was significantly higher in the liver, muscle, and intestine of fish fed LO and SO, compared with the fish fed FO (*p* < 0.05) in large yellow croaker and rainbow trout. There were no significant differences between LO group and SO group in tissues of large yellow croaker and liver of rainbow trout (*p* > 0.05). However, the C/EBPα protein levels in muscle and intestine of rainbow trout were significantly higher in SO group compared with LO group (*p* < 0.05) (Figure 6). The *gata3* expression in intestine and muscle of large yellow croaker was significantly decreased in SO and LO group than FO group (*p* < 0.05) (Figure 5b), but there were no significant differences in liver of large yellow croaker and rainbow trout in SO and LO group compared with FO group (*p* > 0.05) (Figure 5c).

### 3.7. Effects of RNA Interference on Expression of c/ebpα, gata3, and Its Potential Target Genes fads2

**Figure 7:**
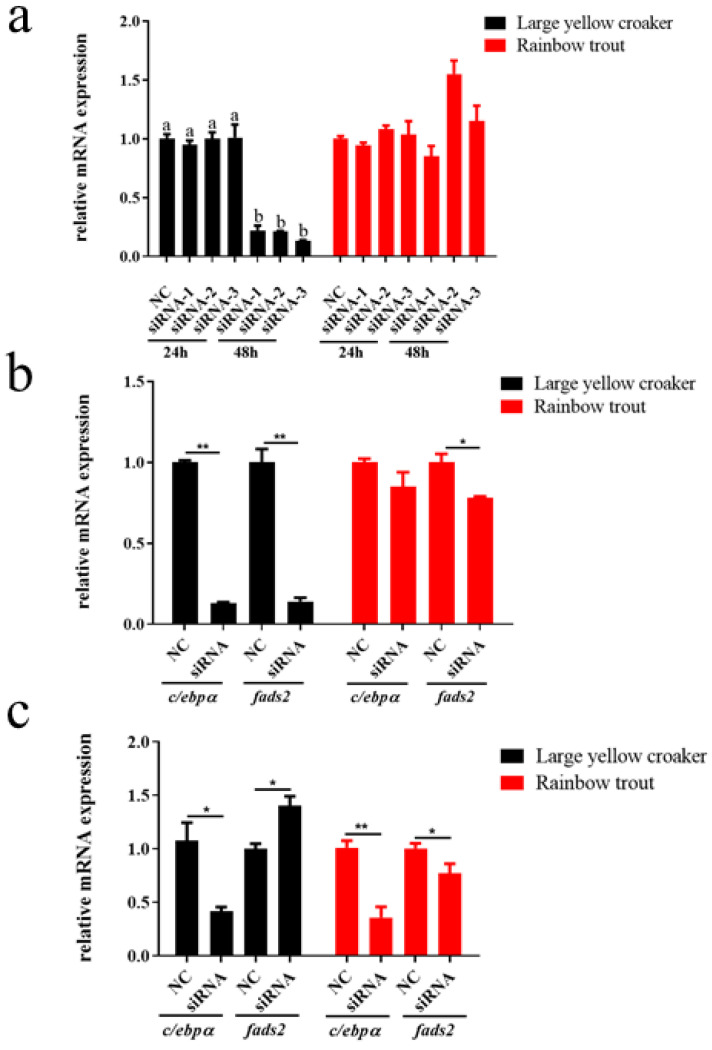
Effects of small RNAi on expression of *c/ebpα*, *gata3*, and its potential target genes *fads2.* Selection of siRNA to target the C/EBPα gene at different time points (**a**), the relative expression and *fads2* under the influence of siRNA-1 in rainbow trout and siRNA-3 in large yellow croaker for 48 h (**b**), the relative expression of *gata3* and *fads2* under the influence of siRNA in rainbow trout and large yellow croaker for 24 h (**c**). Values are presented as mean ± SEM (*n* = 3). Bars without sharing a common letter are significantly different (*p* < 0.05). The “*” means a significant difference (*p* < 0.05) and “**” means a highly significant difference (*p* < 0.01).

To investigate whether C/EBPα and GATA3 can directly affect the expression of *fads2*, we conducted the RNAi assay. The results showed that interference with siRNA-1 in rainbow trout and siRNA-3 in large yellow croaker for 48 h decreased the expression of *c/ebpα* mostly (Figure 7a). The interference of *c/ebpα* expression by these siRNA significantly decreased the expression of *fads2* (*p* < 0.05) in rainbow trout and large yellow croaker (Figure 7b). The interference of *gata3* expression significantly decreased the expression of *fads2* in rainbow trout, but significantly increased the expression of *fads2* in large yellow croaker (Figure 7c).

### 3.8. Site-Directed Mutation of C/EBPα and GATA3 Binding Sites 

**Figure 8:**
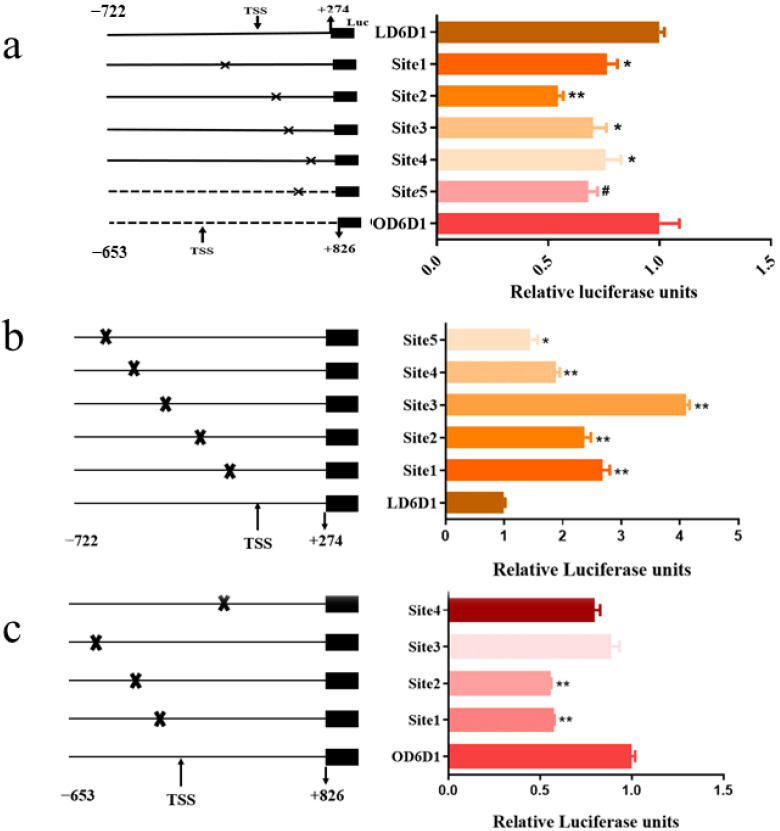
Site-directed mutation of C/EBPα and GATA3 binding sites. The effect of C/EBPα site-directed mutation on large yellow croaker and rainbow trout *fads2* promoter activity (**a**). The effect of GATA3 site-directed mutation on large yellow croaker (**b**) and rainbow trout (**c**) *fads2* promoter activity. Each constructed site-directed mutant was co-transfected with C/EBPα or GATA3 over-expression plasmids and phRL-CMV, and the promoter activity normalized to corresponding non-mutated promoter, LD6D1 and OD6D1. Values are presented as mean ± SEM (*n* = 3). TSS: transcription start site. The “*” and “#” means a significant difference (*p* < 0.05) and “**” means a highly significant difference (*p* < 0.01).

In order to further investigate the function of C/EBPα and GATA3 on the *fads2* promoters of large yellow croaker and rainbow trout, bioinformatics analysis of preliminary identification of C/EBPα and GATA3 binding sites in the promoter was conducted through online software, The JASPAR database and Gene Regulation. Four and one C/EBPα binding sites, and five and four GATA3 binding sites were predicted within the promoters of large yellow croaker and rainbow trout, respectively. The putative binding sites were mutated (Supplementary Figure S1) and detected. The results show that mutation of C/EBPα binding sites causes a significant decrease in transcriptional activity compared to the wild type LD6D1 (*fads2* promoter of large yellow croaker) and OD6D1 (*fads2* promoter of rainbow trout) (*p* < 0.05) (Figure 8a). Mutation of GATA3 binding sites caused a significant increase compared with LD6D1 (*p* < 0.05) (Figure 8b), but a significant decrease compared with OD6D1 (*p* < 0.05) (Figure 8c).

### 3.9. Identification of C/EBPα and GATA3 Binding Sites in the fads2 Promoter Region

**Figure 9:**
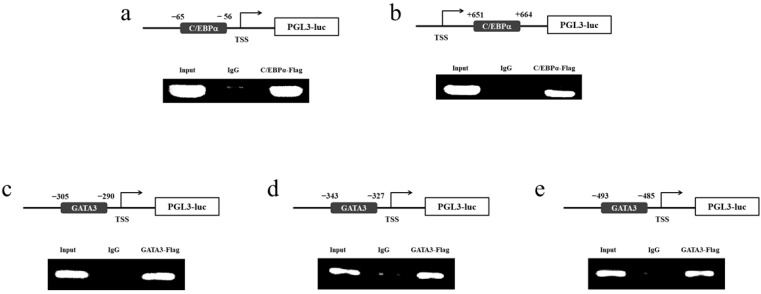
ChIP assay. Identification of C/EBPα binding sites in region of the *fads2* promoter in large yellow croaker (**a**) and rainbow trout (**b**), identification of GATA3 binding sites in region of the *fads2* promoter in large yellow croaker (**c**) and rainbow trout (**d**,**e**).

**Figure 10:**
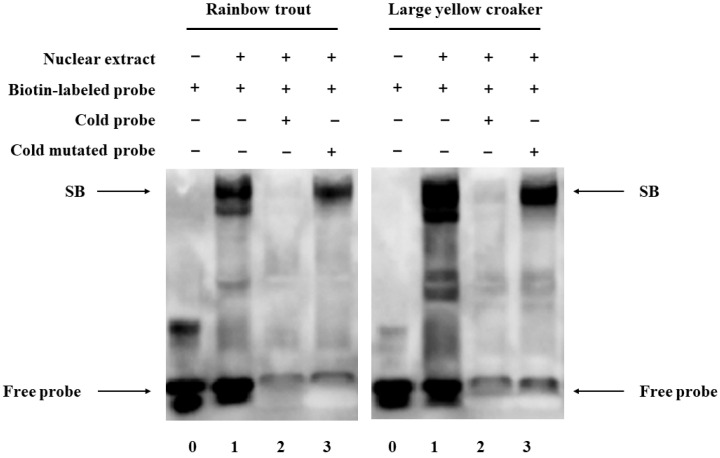
EMSA assay. EMSA assay to identify the binding of C/EBPα and *fads2* promoter in rainbow trout and large yellow croaker.”+” means present; “−“ means absent.

To determine whether the *fads2* promoter is a direct target of C/EBPα and GATA3, we conducted the ChIP assay. The results showed a significant binding of C/EBPα and GATA3 to the promoter of *fads2*. No DNA fragment was amplified from the precipitate by control IgG, indicating the specific interaction between C/EBPα and GATA3 with the *fads2* promoter (Figure 9). The EMSA results also indicate that C/EBPα can bind to the promoter of *fads2* (Figure 10).

## 4. Discussion

The LC-PUFA relative content in the liver of large yellow croaker fed an LO diet was significantly decreased than the fish fed an FO diet, but there was no difference in the liver of rainbow trout, suggesting that rainbow trout had a greater capability to biosynthesis LC-PUFA compared with large yellow croaker. The biosynthesis of LC-PUFA was dependent on dietary C18:3n-3 and C18:2n-6 via desaturases and elongases [6,7,42,43,44], in which Δ6Fads2 was the first rate-limiting enzyme [45,46]. The expression of *fads2* was subject to nutritional regulation and could be up-regulated in fish fed a vegetable oil diet compared to fish fed a fish oil diet [47,48]. The results in this study suggested that 100% replacement of fish oil with vegetable oil caused the increase of *fads2* expression in the liver and intestine of large yellow croaker and rainbow trout, consistent with other studies [31,45,46,48]. 

It was consistent with previous studies that marine fish species show less ability to synthesize LC-PUFA from C18:2n-6 and C18:3n-3 [10,11,48,49,50,51], and the Δ6-desaturase activities vary in different fish species; generally, freshwater fish and salmonid fish show higher Δ6-desaturase activities than marine fish [52]. Our results also showed that the *fads2* expression was higher in rainbow trout than in large yellow croaker, whether with the fish oil diet or the vegetable oil diet. The expression level of *fads2* generally indicates the capacity of LC-PUFA synthesis in fish [45,53]. These results might explain the reasons why large yellow croaker had a low ability to synthesize LC-PUFA, which was consistent with other studies [52]. Another important reason was the lack of Δ5-desaturation capability [54,55].

In recent years, the understanding of the *fads2* promoter in fish had gradually advanced [17,31,52], but the underlying mechanism has not been well-researched. In the present study, deletion mutation of the *fads2* promoter and dual-luciferase reporter assay was carried out to identify the core promoter of *fads2*. Two fragments in large yellow croaker and 1 fragment in rainbow trout were identified as the core promoters. Based on the results, it was different from other studies in which just one core promoter was identified [52,56], indicating the complexity of regulation of gene transcription. Many studies showed that the transcriptional regulation of desaturase genes was regulated by relevant transcription factors [31,56,57,58]. SREBP-1 and PPARα could regulate the transcription of *fads2* that are subjected to dietary fatty acids in large yellow croaker, rainbow trout, and Japanese sea bass [31]. HNF4α was identified as a transcription factor that can bind to the core promoter of Δ4Fads2 and regulate the expression of Δ4Fads2 in rabbitfish [56]. LXR and PPARγ can promote the expression of Δ4Fads2 and Δ5/Δ6Fads2 in rabbitfish [57]. NF-1 and HNF4α can bind to the Δ5/Δ6Fads2 promoter to regulate the expression of Δ5/Δ6Fads2 [58]. We found that the activity of the rainbow trout *fads2* promoter was significantly higher than large yellow croaker, which may be caused by the different regulation of transcription factors. In this study, several transcription factors predicted by JASPAR and Gene Regulation database were used to test the regulation effects on the *fads2* promoter. The results showed that different transcription factors played different functions, in which C/EBPα and GATA3 played an important role. 

Previous studies have shown that C/EBPα could be involved in the process of fatty acid and lipid metabolism by regulating some gene transcription, such as FABP1 [27], FAS, and ACC [28]. C/EBPα is acetylated by p300 and deacetylated by SIRT1, which is a key mediator of SIRT1-controlled adaption of energy homeostasis to changes in nutrient supply [59]. C/EBPα was also important in adipocyte differentiation and adipogenesis [60,61,62,63,64,65]. GATA3 was an important biomarker in development and disease [30]. This study showed that GATA3 binds to regulatory elements and controls target gene expression through physical interaction with core promoter regions [66]. In T cell lineages, many genes were either positively or negatively regulated by GATA3 in a cell type-specific manner, suggesting that GATA3-mediated gene regulation depends strongly on cofactors existing in different T cells [67]. However, the role of C/EBPα and GATA3 in LC-PUFA biosynthesis and the transcription regulation has not been studied. Here, we first showed its functions in regulating LC-PUFA biosynthesis. The results suggested that C/EBPα, *gata3*, and *fads2* have a correlation in response to the dietary fatty acid composition. Further site-directed mutation, ChIP assay, EMSA assay, and siRNA assay showed that C/EBPα and GATA3 could bind to the *fads2* promoter and regulate the transcription directly, also that C/EBPα and GATA3 could regulate the expression of *fads2*.

In conclusion, this study identified the core promoters of *fads2* in large yellow croaker and rainbow trout and suggested that C/EBPα and GATA3 are powerful transcription factors in the regulation of *fads2* transcription. Furthermore, the different regulation of different transcription factors may be one of the reasons for the difference in *fads2* expression in large yellow croaker and rainbow trout. This research took C/EBPα and GATA3 as transcription factors involved in fatty acid biosynthesis in vertebrates and may help us to have a comprehensive understanding of LC-PUFA biosynthesis.

## Figures and Tables

**Table 1:** The polyunsaturated fatty acid composition in diet, liver, muscle, and intestine of large yellow croaker (% total fatty acid methyl esters).

Fatty Acids	Diet [41]				Liver [41]				Muscle [41]				Intestine		
	^1^FO	^2^LO	^3^SO		FO	LO	SO		FO	LO	SO		FO	LO	SO
18:2n-6	10.16	20.31	47.92		7.16 ± 0.76 ^a^	14.6 ± 1.59 ^b^	34.25 ± 1.89 ^c^		7.80 ± 0.23 ^a^	13.21 ± 0.28 ^b^	28.09 ± 0.58 ^c^		9.07 ± 0.39 ^a^	22.04 ± 0.94 ^b^	47.13 ± 0.80 ^c^
18:3n-3	2.04	44.08	4.76		1.18 ± 0.09 ^a^	19.25 ± 1.62 ^b^	2.43 ± 0.27 ^a^		1.34 ± 0.08 ^a^	14.90 ± 0.42 ^c^	2.40 ± 0.08 ^b^		0.98 ± 0.09 ^a^	31.29 ± 0.63 ^c^	3.62 ± 0.62 ^b^
20:4n-6	0.83	-	-		0.47 ± 0.05 ^b^	0.11 ± 0.02 ^a^	0.13 ± 0.03 ^a^		0.76 ± 0.09 ^b^	0.67 ± 0.08 ^ab^	0.45 ± 0.06 ^a^		2.70 ± 0.21 ^b^	0.45 ± 0.1 ^a^	0.35 ± 0.08 ^a^
EPA	6.4	-	0.16		2.03 ± 0.13 ^b^	0.16 ± 0.03 ^a^	0.14 ± 0.02 ^a^		3.92 ± 0.09 ^b^	1.60 ± 0.07 ^a^	1.34 ± 0.09 ^a^		4.76 ± 0.14 ^b^	0.38 ± 0.05 ^a^	0.61 ± 0.1 ^a^
DHA	8.64	-	-		2.89 ± 0.37 ^b^	0.26 ± 0.08 ^a^	0.16 ± 0.01 ^a^		6.85 ± 0.46 ^b^	3.99 ± 0.26 ^a^	3.01 ± 0.35 ^a^		14.63 ± 0.43 ^b^	1.16 ± 0.25 ^a^	0.83 ± 0.27 ^a^
EPA + DHA	15.04	-	0.16		4.92 ± 0.49 ^b^	0.41 ± 0.08 ^a^	0.43 ± 0.15 ^a^		10.77 ± 0.46 ^b^	5.58 ± 0.31 ^a^	4.35 ± 0.44 ^a^		19.39 ± 0.46 ^b^	1.77 ± 0.35 ^a^	1.20 ± 0.31 ^a^

Note: The table just gives the data of polyunsaturated fatty acids that this study mainly paid attention to (The full fatty acid composition data were in Supplementary Tables S5–S11). The values of fatty acids in liver, muscle, and intestine are presented as mean ± SEM (*n* = 3). Values in the same row of each group in each tissue without sharing a common letter are significantly different (*p* < 0.05). The fatty acid compositon of diet, liver, and muscle were data from the previous study [41]. ^1^FO: 100% fish oil as a lipid source in the diet. ^2^LO: 100% linseed oil as a lipid source in the diet. ^3^SO: 100% soybean oil as a lipid source in the diet.

**Table 2:** The polyunsaturated fatty acid composition in liver, muscle, and intestine of rainbow trout (% total fatty acid methyl esters).

Fatty Acids	Liver				Muscle				Intestine		
	^1^FO	^2^LO	^3^SO		FO	LO	SO		FO	LO	SO
18:2n-6	2.54 ± 0.13 ^a^	4.55 ± 0.21 ^b^	9.82 ± 0.63 ^c^		6.56 ± 0.10 ^a^	11.24 ± 0.24 ^b^	24.85 ± 0.58 ^c^		4.61 ± 0.36 ^a^	10.88 ± 0.17 ^b^	19.28 ± 0.11 ^c^
18:3n-3	0.11 ± 0.06 ^a^	2.63 ± 0.12 ^b^	0.28 ± 0.04 ^a^		1.03 ± 0.01 ^a^	15.33 ± 0.45 ^b^	1.37 ± 0.11 ^a^		0.61 ± 0.01 ^a^	13.80 ± 0.26 ^b^	0.96 ± 0.13 ^a^
20:4n-6	1.43 ± 0.21 ^a^	1.57 ± 0.44 ^a^	4.49 ± 0.48 ^b^		0.92 ± 0.14 ^b^	0.37 ± 0.03 ^a^	1.57 ± 0.10 ^c^		2.00 ± 0.10 ^a^	1.25 ± 0.27 ^a^	3.67 ± 0.57 ^b^
EPA	1.08 ± 0.23 ^b^	1.98 ± 0.15 ^c^	0.18 ± 0.02 ^a^		2.80 ± 0.14 ^c^	1.62 ± 0.07 ^b^	0.53 ± 0.04 ^a^		1.75 ± 0.17	1.38 ± 0.10	-
DHA	17.58 ± 3.24 ^b^	15.12 ± 0.20 ^b^	4.58 ± 0.63 ^a^		12.16 ± 1.67 ^b^	4.61 ± 0.32 ^a^	2.88 ± 0.31 ^a^		16.40 ± 1.65 ^b^	7.74 ± 0.87 ^a^	4.03 ± 0.61 ^a^
EPA + DHA	18.66 ± 3.46 ^b^	17.10 ± 0.05 ^b^	4.76 ± 0.66 ^a^		14.96 ± 1.81 ^b^	6.23 ± 0.38 ^a^	3.41 ± 0.34 ^a^		18.15 ± 1.73 ^c^	9.12 ± 0.77 ^b^	4.19 ± 0.62 ^a^

Note: The values of fatty acids in liver, muscle, and intestine are presented as mean ± SEM (*n* = 3). Values in the same row of each group in each tissue without sharing a common letter are significantly different (*p* < 0.05). ^1^FO: 100% fish oil as a lipid source in the diet. ^2^LO: 100% linseed oil as a lipid source in the diet. ^3^SO: 100% soybean oil as a lipid source in the diet.

## Data Availability

Not applicable.

## Supplementary Materials

The following supporting information can be downloaded at: https://www.mdpi.com/article/10.3390/biom12050659/s1, Figure S1: Site-directed mutation of C/EBPα (A) and GATA3 binding sites on Δ6Fad promoter in large yellow croaker (B) and rainbow trout (C). The letters shade in gray were the binding sites of C/EBPα and GATA3, the letters underlined were the mutated sites; Table S1: Formulation and proximate composition of the experimental diets (% dry matter); Table S2: Sequences of the PCR primers used in this study for qPCR analysis and ChIP; Table S3: Sequences of the PCR primers used in this study for fads2 promoter deletion cloning of large yellow croaker and rainbow trout; Table S4: Sequences of the PCR primers used in this study for site-directed mutation; Table S5: Fatty acid composition of diets (% total fatty acid methyl esters); Table S6: Fatty acid composition in liver of large yellow croaker (% total fatty acid methyl esters); Table S7: Fatty acid composition in muscle of large yellow croaker (% total fatty acid methyl esters); Table S8: Fatty acid composition in intestine of large yellow croaker (% total fatty acid methyl esters); Table S9: Fatty acid composition in liver of rainbow trout (% total fatty acid methyl esters); Table S10: Fatty acid composition in muscle of rainbow trout (% total fatty acid methyl esters); Table S11: Fatty acid composition in intestine of rainbow trout (% total fatty acid methyl esters).

## Abbreviation

Δ6Fads2: Δ6 fatty acyl desaturase; C/EBPα, CCAAT/enhancer-binding protein α; GATA3, GATA binding protein 3; LC-PUFA, long-chain polyunsaturated fatty acids; EPA, eicosapentaenoic acid; DHA, docosahexaenoic acid; ARA, arachidonic acid; LA, linoleic acid; ALA, α-linolenic acid; FO, fish oil; LO, linseed oil; SO, soybean oil; EMSA, electrophoretic mobility shift assay; ChIP, chromatin immunoprecipitation; siRNA, small interference RNA.
