# Supplementary material for: Regulation of Δ6Fads2 Gene Involved in LC-PUFA Biosynthesis Subjected to Fatty Acid in Large Yellow Croaker (Larimichthys crocea) and Rainbow Trout (Oncorhynchus mykiss)
Source: Biomolecules. 2022 Apr 30;12(5):659. doi: 10.3390/biom12050659 (PMC9139026; doi:10.3390/biom12050659)
Supplement: Supplementary file 1 [file biomolecules-12-00659-s001.zip › biomolecules-1669543-supplementary.pdf]

biomolecules

Supplementary materials

Table S1. Formulation and proximate composition of the experimental diets (% dry matter) [41].

Ingredient	Fish oil	Linseed oil	Soybean oil
Casein	36.80	36.80	36.80
Gelatin	9.20	9.20	9.20
Dextrin	28.00	28.00	28.00
Microcrystalline cellulose	3.00	3.00	3.00
α-starch	6.35	6.35	6.35
Lecithin	2.00	2.00	2.00
Vitamin premix	2.00	2.00	2.00
Mineral salt	2.00	2.00	2.00
Antioxidant	0.05	0.05	0.05
Attractant mixture[1]	0.30	0.30	0.30
Mold inhibitor[2]	0.10	0.10	0.10
Choline chloride	0.20	0.20	0.20
Fish oil	10.00	0.00	0.00
Linseed oil	0.00	10.00	0.00
Soybean oil	0.00	0.00	10.00
Total	100.00	100.00	100.00
Proximate composition (% dry matter)			
Crude protein	43.38	43.95	42.57
Crude lipid	11.23	12.08	11.32

[1]Attractant compromise 25% glycine and 75% betaine.
[2] Mold inhibitor compromise 50% Calcium and 50% Fumaric acid.

Table S2. Sequences of the PCR primers used in this study for qPCR analysis and ChIP.

Species	Primer	Sequences5′-3′
Large yellow croaker	Δ6Fad-F	CGCTTCCTCTGCTGCTATG
	Δ6Fad-R	TCCTGGGAAACAAATGGTG
	C/EBPα-F	GGAGACGCAGCAGAAGGTG
	C/EBPα-R	TGCCCAGTGGTTTGAAGGA
	GATA3-F	CAGTCCGCACCTCTTCACG
	GATA3-R	CGATTGATGCCACGCTCC
	β-actin-F	GACCTGACAGACTACCTCATG
	β-actin-R	AGTTGAAGGTGGTCTCGTGGA
	C/EBPα ChIP-F	TGTGAGTGAATCCCGAAGG
	C/EBPα ChIP-R	GCATCATTTGTCTGTTTCG
	GATA3 ChIP-F	GCCACACGATATCTCCGATTATT
	GATA3 ChIP-R	GCAAGGAAAAAAATCTATATTTAGCCT
Rainbow trout	Δ6Fad-F	ATGACTACGGCCACCTGTC
	Δ6Fad-R	TGGTGCTGGAAGTGACGAT
	C/EBPα-F	GCAGGGCTATGGCTGTATT
	C/EBPα-R	GCTGGTGGGAATGGTGGTA
	GATA3-F	GGACGGAAGCAAAGCAAT
	GATA3-R	GACGCCGAAGGGTAGACA
	β-actin-F	ATCAGGGAGTGATGGTTGGGATG
	β-actin-R	CTCGTAGATGGGTACTGTGTGGG
	C/EBPα ChIP-F	GCAGAAGAATCGCAGGTTA
	C/EBPα ChIP-R	GAGGAAGTCAGGGAATGGA
	GATA3 ChIP-F1	ACCATAGTGAGCTACAGTTAGTTATGTAACG
	GATA3 ChIP-R1	AAATTAAATGATACAATGATGATGTGGA
	GATA3 ChIP-F2	AAAGATTTGCACTGTGTGTCATATAAAA
	GATA3 ChIP-R2	CAAAGTTTATTTATATCTAATAACGAAAACCT

Table S3. Sequences of the PCR primers used in this study for *fads2* promoter deletion cloning of large yellow croaker and rainbow trout.

**Species**	**Primer**	**Sequences5′-3′**
Large yellow croaker	LD6D1F	GCCGGTACCTGTGTATAATGGAGC
	LD6D2F	CGGGGTACCGATTGAGCCTATTGCAC
	LD6D3F	CGGGGTACCTAATGTGAGTGAATCC
	LD6D4F	CGGGGTACCAATGAGTGGGTGAATG
	LD6D5F	CGGGGTACCTACTATGCATTGCAAC
	LD6D6F	CGGGGTACCTGTCGAAACAGACAAAT
	LD6D7F	CGGGGTACCTTGTCTTACTTCACTTGT
	R	CCCAAGCTTCCTCACTGCTGTCTCT
Rainbow trout	OD6D1F	TAGGTACCAGACGGGGCTGTCCTAT
	OD6D2F	CGGGGTACCAATTGTTTAGGGAACTTGAG
	OD6D3F	CGGGGTACCCTGTATCCACATCATCATT
	OD6D4F	CGGGGTACCAGAAGAGGAGTCCATGC
	OD6D5F	CGGGGTACCACAGGTTTTCGTTATT
	OD6D6F	CGGGGTACCAATCGCAGGTTAG
	OD6D7F	CGGGGTACCGACAAGACTCATCCC
	R	CCAAGCTTCCTCGGTTCTCTCTGC
Standard linear plasmid construction	LDF	TTCGCTTCCTCTGCTGCTA
	LDR	TGCTAACTTTGTGCCCTGT
	ODF	CTGAAGCCGTTGCTGATTG
	ODR	ATCCACCCAGTTCCGTTGT

Notes: Restriction sites underlined are *HindIII* (AAGCTT) and *KpnI* (GGTACC) in *fads2* reporter vector construction. F means forward primer. R means reverse primer.

Table S4. Sequences of the PCR primers used in this study for site-directed mutation.

Primer		Sequences5′-3′
C/EBPα	Site1	F: TCTCCGATTAGGTTTAATTAATCGATACG
		R: AAACCTAATCGGAGATATCGTGTGGCAGGC
	Site2	F: GCTTCATGTCACGACAGACAAATGATGCCAAC
		R: GTCGTGACATGAAGCATGTATATAAAAGCC
	Site3	F: GTTGTCGGATGGTATCATCAATTATTAGTG
		R: ATACCATCCGACAACCAGGAGGCTGTTGAT
	Site4	F: TGAAGATGCGTGTCAGCTCTTTGTCTTACT
		R: TGACACGCATCTTCACTAATAATTGATGAT
	Site5	F: CACACGAGCTATCTACGTAGGTGTGACTTG
		R: GTAGATAGCTCGTGTGACTCTTCTACAGTT
GATA3 of large yellow croaker	Site1	F: TATTTTTCCTTGCCTCTGAATTGAATCATCTGG
		R: CAGAGGCAAGGAAAAATATTTAGCCTGTAAATGCGTTTTCA
	Site2	F: CCTGCCACACGATATTGTTTAATTAATCGATACGCAGG
		R: ACAATATCGTGTGGCAGGCTGTTCAGCCTACAG
	Site3	F: TGGATGAAACAGGAAGGCATAAAGTGATCCGGC
		R: GCCTTCCTGTTTCATCCATTTTAATTCGTTAACACCC
	Site4	F: CTGTACTCGCTCAGAAACCCTCGAATGATCGGC
		R: GTTTCTGAGCGAGTACAGTACATGCTGGACCACTG
	Site5	F: TCAGATTGAGCCTATTGCACATCAGCCAGTGGT
		R: GCAATAGGCTCAATCTGAATATTACAGAGTCGTCTGAGCG
GATA3 of rainbow trout	Site1	F: GCCTACTGGTGATAGATGATGAGACGGGTGATCAGA
		R: CATCTATCACCAGTAGGCTACTAACGGTATATATC
	Site2	F: AATTGCCAGCGGTCTAGTGAAATAACCCCATTG
		R: ACTAGACCGCTGGCAATTGGTTCACAACAGCCT
	Site3	F: GAGCGTCCGTTGAAATAGGCAAGCCTTTGACAG
		R: CTATTTCAACGGACGCTCTTGGACGCCCTCACG
	Site4	F: GCTCCACATAGAAACTCCATACAGAATGCTACAGG
		R: GGAGTTTCTATGTGGAGCATCGATGCAATTTACC

Table S5. Fatty acid composition of diets (% total fatty acid methyl esters) [41].

Fatty acids	Diets		
	**FO**	**LO**	**SO**
14:0	5.44	0.46	0.60
16:0	21.30	9.26	13.00
18:0	4.54	4.82	4.54
20:0	0.61	-	0.39
∑SFA	31.89	14.54	18.53
16:1n-7	5.85	-	0.29
18:1n-9	16.56	21.07	26.44
18:1n-7	3.16	-	-
20:1n-9	2.53	-	0.41
∑MUFA	28.10	21.07	27.14
18:2n-6	10.16	20.31	47.92
20:4n-6	0.83	-	-
∑n-6PUFA	10.99	20.31	47.92
18:3n-3	2.04	44.08	4.76
20:5n-3	6.40	-	0.16
22:6n-3	8.64	-	-
∑n-3PUFA	17.08	44.08	4.92
n-3/n-6PUFA	1.55	2.17	0.10
∑n-3LC-PUFA	15.04	-	0.16
DHA/EPA	1.35	-	-

Note: "-" means the fatty acid was undetected; SFA: saturated fatty acids; MUFA: monounsaturated fatty acids; PUFA: polyunsaturated fatty acids; LC-PUFA: long chain-polyunsaturated fatty acids. FO: 100% fish oil as a lipid source in the diet. LO: 100% linseed oil as a lipid source in the diet. SO: 100% soybean oil as a lipid source in the diet.

Table S6. Fatty acid composition in liver of large yellow croaker (% total fatty acid methyl esters) [41].

Fatty acids	Diets		
	FO	LO	SO
14:0	2.84 ± 0.14[b]	0.81 ± 0.11[a]	0.85 ± 0.12[a]
16:0	24.00 ± 0.51[b]	8.97 ± 0.25[a]	10.56 ± 1.57[a]
18:0	8.00 ± 0.49	8.24 ± 1.49	8.03 ± 1.30
20:0	0.24 ± 0.01[b]	0.18 ± 0.02[a]	0.23 ± 0.02[ab]
∑SFA	35.09 ± 0.26[b]	18.21 ± 1.69[a]	19.68 ± 2.18[a]
16:1n-7	9.47 ± 0.16[b]	3.64 ± 0.56[a]	3.02 ± 0.60[a]
18:1n-9	28.46 ± 0.94[a]	37.68 ± 2.86[b]	34.79 ± 1.09[ab]
18:1n-7	2.74 ± 0.02[c]	1.37 ± 0.05[a]	1.62 ± 0.01[b]
20:1n-9	2.00 ± 0.01[b]	0.98 ± 0.06[a]	0.80 ± 0.08[a]
∑MUFA	42.67 ± 1.00	43.67 ± 3.48	40.23 ± 1.63
18:2n-6	7.16 ± 0.76[a]	14.63 ± 1.59[b]	34.25 ± 1.89[c]
20:4n-6	0.47 ± 0.05[b]	0.11 ± 0.02[a]	0.13 ± 0.03[a]
∑n-6PUFA	7.63 ± 0.78[a]	14.75 ± 1.61[b]	34.39 ± 1.93[c]
18:3n-3	1.18 ± 0.09[a]	19.25 ± 1.62[b]	2.43 ± 0.27[a]
20:5n-3	2.03 ± 0.13[b]	0.16 ± 0.03[a]	0.14 ± 0.02[a]
22:6n-3	2.89 ± 0.37[b]	0.26 ± 0.08[a]	0.16 ± 0.01[a]
∑n-3PUFA	6.10 ± 0.53[a]	19,67 ± 1.65[b]	2.86 ± 0.01[a]
n-3/n-6PUFA	0.81 ± 0.09[b]	1.37 ± 0.18[c]	0.08 ± 0.01[a]
∑n-3LC-PUFA	4.92 ± 0.49[b]	0.41 ± 0.08[a]	0.43 ± 0.15[a]
DHA/EPA	1.41 ± 0.11	1.68 ± 0.11	1.96 ± 0.66

Notes: "-" means the fatty acid was undetected; SFA: saturated fatty acids; MUFA: monounsaturated fatty acids; PUFA: polyunsaturated fatty acids; LC-PUFA: long chain-polyunsaturated fatty acids. FO: 100% fish oil as a lipid source in the diet. LO: 100% linseed oil as a lipid source in the diet. SO: 100% soybean oil as a lipid source in the diet. The values are presented as mean ± SEM (n = 3). Values in the same row of each group without sharing a common letter are significantly different ($P < 0.05$).

Table S7. Fatty acid composition in muscle of large yellow croaker (% total fatty acid methyl esters) [41].

Fatty acids	Diets		
	FO	LO	SO
14:0	3.30 ± 0.16[b]	2.10 ± 0.03[a]	1.94 ± 0.13[a]
16:0	24.13 ± 0.44[b]	18.17 ± 0.03[a]	17.63 ± 0.10[a]
18:0	6.65 ± 0.26[ab]	7.34 ± 0.28[b]	6.35 ± 0.21[a]
20:0	0.42 ± 0.02[b]	0.39 ± 0.02[ab]	0.35 ± 0.02[a]
ΣSFA	34.50 ± 0.58[c]	28.00 ± 0.29[b]	26.26 ± 0.10[a]
16:1n-7	6.88 ± 0.21[b]	3.83 ± 0.17[a]	3.66 ± 0.18[a]
18:1n-9	22.21 ± 0.45[a]	24.31 ± 0.82[ab]	26.90 ± 0.99[b]
18:1n-7	2.79 ± 0.08	-	-
20:1n-9	2.25 ± 0.09[c]	1.69 ± 0.02[b]	1.32 ± 0.08[a]
ΣMUFA	34.13 ± 0.55[b]	30.54 ± 0.77[a]	32.59 ± 0.16[b]
18:2n-6	7.80 ± 0.23[a]	13.21 ± 0.28[b]	28.09 ± 0.58[c]
20:4n-6	0.76 ± 0.09[b]	0.67 ± 0.08[ab]	0.45 ± 0.06[a]
Σn-6PUFA	8.56 ± 0.23[a]	13.87 ± 0.35[b]	28.54 ± 0.53[c]
18:3n-3	1.34 ± 0.08[a]	14.90 ± 0.42[c]	2.40 ± 0.08[b]
20:5n-3	3.92 ± 0.09[b]	1.60 ± 0.07[a]	1.34 ± 0.09[a]
22:6n-3	6.85 ± 0.46[b]	3.99 ± 0.26[a]	3.01 ± 0.35[a]
Σn-3PUFA	12.11 ± 0.39[b]	20.48 ± 0.34[c]	6.75 ± 0.37[a]
n-3/n-6PUFA	1.42 ± 0.06[b]	1.48 ± 0.04[b]	0.24 ± 0.02[a]
Σn-3LC-PUFA	10.77 ± 0.46[b]	5.58 ± 0.31[a]	4.35 ± 0.44[a]
DHA/EPA	1.75 ± 0.12[a]	2.50 ± 0.13[b]	2.24 ± 0.11[b]

Notes: "-" means the fatty acid was undetected; SFA: saturated fatty acids; MUFA: monounsaturated fatty acids; PUFA: polyunsaturated fatty acids; LC-PUFA: long chain-polyunsaturated fatty acids. FO: 100% fish oil as a lipid source in the diet. LO: 100% linseed oil as a lipid source in the diet. SO: 100% soybean oil as a lipid source in the diet. The values are presented as mean ± SEM (n = 3). Values in the same row of each group without sharing a common letter are significantly different ($P < 0.05$).

Table S8. Fatty acid composition in intestine of large yellow croaker (% total fatty acid methyl esters).

Fatty acids	Diets		
	FO	LO	SO
14:0	2.27 ± 0.29[b]	0.44 ± 0.04[a]	0.38 ± 0.01[a]
16:0	20.06 ± 1.15[b]	7.75 ± 0.26[a]	9.42 ± 0.24[a]
18:0	12.15 ± 1.15[b]	6.94 ± 0.53[a]	6.20 ± 0.68[a]
20:0	0.44 ± 0.01[b]	0.18 ± 0.01[a]	0.21 ± 0.02[a]
∑SFA	34.92 ± 2.02[b]	15.31 ± 0.77[a]	16.21 ± 0.77[a]
16:1n-7	4.23 ± 0.54[b]	1.57 ± 0.32[a]	1.83 ± 0.40[a]
18:1n-9	14.40 ± 0.51[a]	23.63 ± 1.14[b]	25.36 ± 0.52[b]
18:1n-7	3.70 ± 0.12	-	-
20:1n-9	2.40 ± 0.12[b]	0.83 ± 0.15[a]	0.76 ± 0.04[a]
∑MUFA	24.73 ± 1.08[a]	26.03 ± 1.01[ab]	28.49 ± 0.17[b]
18:2n-6	9.07 ± 0.39[a]	22.04 ± 0.94[b]	47.13 ± 0.80[c]
20:4n-6	2.70 ± 0.21[b]	0.45 ± 0.10[a]	0.35 ± 0.08[a]
∑n-6PUFA	11.77 ± 0.49[a]	22.48 ± 0.93[b]	47.48 ± 0.72[c]
18:3n-3	0.98 ± 0.09[a]	31.29 ± 0.63[c]	3.62 ± 0.62[b]
20:5n-3	4.76 ± 0.14[b]	0.38 ± 0.05[a]	0.61 ± 0.10[a]
22:6n-3	14.63 ± 0.43[b]	1.16 ± 0.25[a]	0.83 ± 0.27[a]
∑n-3PUFA	20.37 ± 0.43[b]	33.05 ± 0.34[c]	4.82 ± 0.45[a]
n-3/n-6PUFA	1.74 ± 0.07[c]	1.48 ± 0.08[b]	0.10 ± 0.01[a]
∑n-3LC-PUFA	19.39 ± 0.46[b]	1.77 ± 0.35[a]	1.20 ± 0.31[a]
DHA/EPA	3.08 ± 0.13[c]	1.87 ± 0.14[a]	2.14 ± 0.48[ab]

Notes: "-" means the fatty acid was undetected; SFA: saturated fatty acids; MUFA: monounsaturated fatty acids; PUFA: polyunsaturated fatty acids; LC-PUFA: long chain-polyunsaturated fatty acids. FO: 100% fish oil as a lipid source in the diet. LO: 100% linseed oil as a lipid source in the diet. SO: 100% soybean oil as a lipid source in the diet. The values are presented as mean ± SEM (n = 3). Values in the same row of each group without sharing a common letter are significantly different ($P < 0.05$).

Table S9. Fatty acid composition in liver of rainbow trout (% total fatty acid methyl esters).

Fatty acids	Diets		
	FO	LO	SO
14:0	2.24 ± 0.20[b]	1.21 ± 0.16[a]	1.40 ± 0.04[a]
16:0	19.68 ± 0.55[b]	19.22 ± 0.31[ab]	17.16 ± 1.08[a]
18:0	4.82 ± 0.53[a]	6.44 ± 0.27[b]	6.65 ± 0.45[b]
20:0	-	-	-
∑SFA	26.74 ± 0.75	26.87 ± 0.41	25.21 ± 1.21
16:1n-7	8.86 ± 1.72	6.38 ± 0.36	5.76 ± 0.58
18:1n-9	26.68 ± 3.05	27.79 ± 2.14	33.17 ± 3.06
18:1n-7	4.34 ± 0.63	-	-
20:1n-9	3.48 ± 0.95	1.61 ± 0.24	2.92 ± 0.26
∑MUFA	43.36 ± 4.79	36.44 ± 2.04	41.85 ± 3.57
18:2n-6	2.54 ± 0.13[a]	4.55 ± 0.21[b]	9.82 ± 0.63[c]
20:4n-6	1.43 ± 0.21[a]	1.57 ± 0.44[a]	4.49 ± 0.48[b]
∑n-6PUFA	3.97 ± 0.32[a]	6.11 ± 0.43[b]	14.31 ± 0.75[c]
18:3n-3	0.11 ± 0.06[a]	2.63 ± 0.12[b]	0.28 ± 0.04[a]
20:5n-3	1.08 ± 0.23[b]	1.98 ± 0.15[c]	0.18 ± 0.02[a]
22:6n-3	17.58 ± 3.24[b]	15.12 ± 0.20[b]	4.58 ± 0.63[a]
∑n-3PUFA	18.78 ± 3.51[b]	19.72 ± 0.06[b]	5.03 ± 0.69[a]
n-3/n-6PUFA	4.72 ± 0.76[b]	3.25 ± 0.20[b]	0.35 ± 0.03[a]
∑n-3LC-PUFA	18.66 ± 3.46[b]	17.10 ± 0.05[b]	4.76 ± 0.66[a]
DHA/EPA	16.73 ± 1.39[b]	7.75 ± 0.68[a]	25.87 ± 1.93[c]

Notes: "-" means the fatty acid was undetected; SFA: saturated fatty acids; MUFA: monounsaturated fatty acids; PUFA: polyunsaturated fatty acids; LC-PUFA: long chain-polyunsaturated fatty acids. FO: 100% fish oil as a lipid source in the diet. LO: 100% linseed oil as a lipid source in the diet. SO: 100% soybean oil as a lipid source in the diet. The values are presented as mean ± SEM (n = 3). Values in the same row of each group without sharing a common letter are significantly different ($P < 0.05$).

Table S10. Fatty acid composition in muscle of rainbow trout (% total fatty acid methyl esters).

Fatty acids	Diets		
	FO	LO	SO
14:0	3.98 ± 0.06[b]	1.69 ± 0.05[a]	1.61 ± 0.04[a]
16:0	22.28 ± 0.39[c]	16.54 ± 0.29[a]	18.65 ± 0.30[b]
18:0	4.39 ± 0.10[a]	4.67 ± 0.06[ab]	4.76 ± 0.12[b]
20:0	-	-	-
∑SFA	30.85 ± 0.40[c]	22.98 ± 0.28[a]	25.14 ± 0.39[b]
16:1n-7	8.65 ± 0.04[c]	5.18 ± 0.06[b]	4.43 ± 0.27[a]
18:1n-9	21.21 ± 0.83[a]	26.49 ± 0.39[b]	27.42 ± 0.95[b]
18:1n-7	3.62 ± 0.09	-	-
20:1n-9	2.29 ± 0.13[b]	1.20 ± 0.17[a]	1.18 ± 0.09[a]
∑MUFA	35.76 ± 0.99[b]	32.87 ± 0.49[a]	33.87 ± 0.92[ab]
18:2n-6	6.56 ± 0.10[a]	11.24 ± 0.24[b]	24.85 ± 0.58[c]
20:4n-6	0.92 ± 0.14[b]	0.37 ± 0.03[a]	1.57 ± 0.10[c]
∑n-6PUFA	7.48 ± 0.11[a]	11.62 ± 0.22[b]	26.42 ± 0.68[c]
18:3n-3	1.03 ± 0.01[a]	15.33 ± 0.45[b]	1.37 ± 0.11[a]
20:5n-3	2.80 ± 0.14[c]	1.62 ± 0.07[b]	0.53 ± 0.04[a]
22:6n-3	12.16 ± 1.67[b]	4.61 ± 0.32[a]	2.88 ± 0.31[a]
∑n-3PUFA	15.99 ± 1.80[b]	21.56 ± 0.08[c]	4.78 ± 0.38[a]
n-3/n-6PUFA	2.13 ± 0.22[b]	1.86 ± 0.03[b]	0.18 ± 0.01[a]
∑n-3LC-PUFA	14.96 ± 1.81[b]	6.23 ± 0.38[a]	3.41 ± 0.34[a]
DHA/EPA	4.31 ± 0.37[b]	2.85 ± 0.12[a]	5.45 ± 0.28[c]

Notes: "-" means the fatty acid was undetected; SFA: saturated fatty acids; MUFA: monounsaturated fatty acids; PUFA: polyunsaturated fatty acids; LC-PUFA: long chain-polyunsaturated fatty acids. FO: 100% fish oil as a lipid source in the diet. LO: 100% linseed oil as a lipid source in the diet. SO: 100% soybean oil as a lipid source in the diet. The values are presented as mean ± SEM (n = 3). Values in the same row of each group without sharing a common letter are significantly different ($P < 0.05$).

Table S11. Fatty acid composition in intestine of rainbow trout (% total fatty acid methyl esters).

Fatty acids	Diets		
	FO	LO	SO
14:0	2.76 ± 0.10[b]	1.21 ± 0.15[a]	1.24 ± 0.09[a]
16:0	25.80 ± 0.47[c]	17.72 ± 0.64[a]	20.43 ± 0.91[b]
18:0	8.78 ± 0.44	7.40 ± 0.62	8.14 ± 0.45
20:0	-	-	-
∑SFA	37.34 ± 0.94[b]	26.38 ± 1.08[a]	29.90 ± 1.18[a]
16:1n-7	5.89 ± 0.25[b]	3.82 ± 0.52[a]	3.24 ± 0.48[a]
18:1n-9	18.67 ± 1.05[a]	25.25 ± 1.28[b]	24.93 ± 1.46[b]
18:1n-7	4.04 ± 0.06	-	2.16 ± 0.10
20:1n-9	2.00 ± 0.01[b]	1.18 ± 0.06[a]	1.21 ± 0.11[a]
∑MUFA	30.60 ± 1.11	31.01 ± 1.13	30.82 ± 1.45
18:2n-6	4.61 ± 0.36[a]	10.88 ± 0.17[b]	19.28 ± 0.11[c]
20:4n-6	2.00 ± 0.10[a]	1.25 ± 0.27[a]	3.67 ± 0.57[b]
∑n-6PUFA	6.61 ± 0.46[a]	12.12 ± 0.34[b]	22.95 ± 0.62[c]
18:3n-3	0.61 ± 0.01[a]	13.80 ± 0.26[b]	0.96 ± 0.13[a]
20:5n-3	1.75 ± 0.17	1.38 ± 0.10	-
22:6n-3	16.40 ± 1.65[b]	7.74 ± 0.87[a]	4.03 ± 0.61[a]
∑n-3PUFA	18.77 ± 1.65[b]	22.93 ± 0.93[c]	5.15 ± 0.57[a]
n-3/n-6PUFA	2.89 ± 0.41	1.89 ± 0.07	0.23 ± 0.03
∑n-3LC-PUFA	18.15 ± 1.73[c]	9.12 ± 0.77[b]	4.19 ± 0.62[a]
DHA/EPA	9.49 ± 0.10	5.74 ± 1.08	-

Notes: "-" means the fatty acid was undetected; SFA: saturated fatty acids; MUFA: monounsaturated fatty acids; PUFA: polyunsaturated fatty acids; LC-PUFA: long chain-polyunsaturated fatty acids. FO: 100% fish oil as a lipid source in the diet. LO: 100% linseed oil as a lipid source in the diet. SO: 100% soybean oil as a lipid source in the diet. The values are presented as mean ± SEM (n = 3). Values in the same row of each group without sharing a common letter are significantly different ($P < 0.05$).

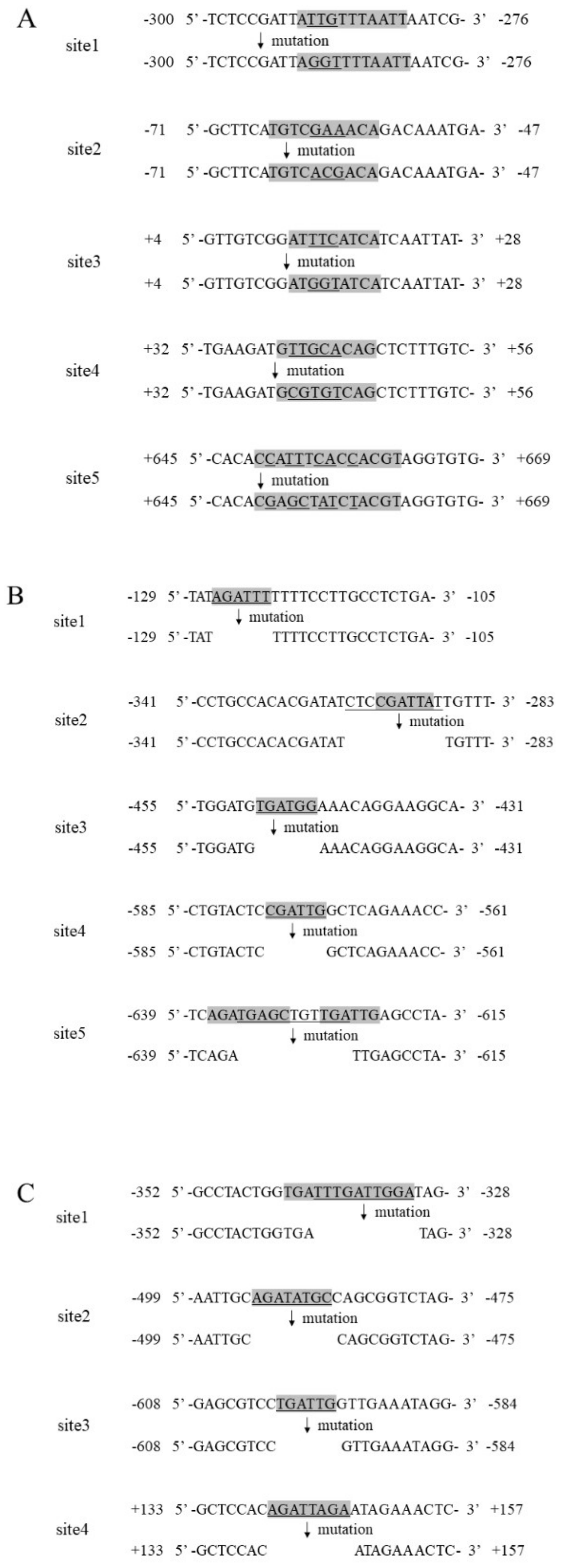

Figure S1. Site-directed mutation of C/EBPα (A) and GATA3 binding sites on Δ6Fad promoter in large yellow croaker (B) and rainbow trout (C). The letters shade in gray were the binding sites of C/EBPα and GATA3, the letters underlined were the mutated sites.
